# Bone marrow adipose tissue expansion and bone loss in experimental chronic kidney disease is independent of altered bone marrow stromal cell lineage determination

**DOI:** 10.3389/fendo.2025.1666681

**Published:** 2025-09-22

**Authors:** Worachet Promruk, William P. Cawthorn, Lucie E. Bourne, Soher N. Jayash, Aine Pears, Katherine A. Staines, Louise A. Stephen, Colin Farquharson

**Affiliations:** ^1^ Functional Genetics Division, The Roslin Institute and Royal (Dick) School of Veterinary Studies, University of Edinburgh, Edinburgh, United Kingdom; ^2^ Chulabhorn Royal Academy, Bangkok, Thailand; ^3^ Institute for Neuroscience and Cardiovascular Research, Edinburgh Bioquarter, University of Edinburgh, Edinburgh, United Kingdom; ^4^ Centre for Lifelong Health, School of Applied Sciences, University of Brighton, Brighton, United Kingdom

**Keywords:** chronic kidney disease, renal osteodystrophy, bone marrow adipocytes, adipokines, bone, corticosteroids

## Abstract

**Introduction:**

Chronic kidney disease-mineral bone disorder is the irreversible loss of kidney function leading to altered mineral homeostasis and bone loss, commonly referred to as renal osteodystrophy. Bone marrow adipose tissue (BMAT) accumulates in clinical CKD and animal models of this disease, but the mechanism(s) responsible are unclear. This study sought to determine the relationship between BMAT distribution and bone structure and to establish whether disease progression directly affected: 1) the commitment of bone marrow mesenchymal stromal cells (BMSCs) to osteoblastic (OPC) and adipogenic (APC) precursor cells, and 2) the differentiation of BMSCs to mature adipocytes and osteoblasts.

**Methods:**

Eight-week-old male C57BL/6J mice received a diet supplemented with 0.2% adenine for ≤5 weeks to induce CKD. Control mice received the same diet without adenine. Serum biochemistries were quantified using a biochemistry analyzer and plasma hormone levels by ELISA. Bone phenotypes were evaluated by µCT. The same bones were decalcified and stained with 1% osmium tetroxide and BMAT quantified using µCT. Precursor cell populations in bone marrow were quantified by flow cytometry.

**Results:**

The development of CKD during the early stages of the disease was confirmed by elevated serum concentrations of blood urea nitrogen and creatinine from 3-weeks’ induction. After 5-weeks’ induction, trabecular bone microarchitecture including bone mineral density was compromised whereas cortical bone area and thickness were decreased in CKD tibiae after 3- and 5-weeks’ induction. Compared to age-matched controls, proximal tibial BMAT tended to increase in CKD mice by 3 weeks’ induction and this reached statistical significance after 5-weeks where there was a negative correlation between regulated BMAT accumulation and trabecular bone loss. BMAT accumulation was not due to calorie deficiency and was positively correlated with circulating adiponectin, but not with circulating leptin or corticosterone. During CKD onset (weeks 1-2) of CKD, BMSCs from CKD mice had enhanced adipogenic potential but the proportions of OPCs and APCs within the bone marrow were unchanged.

**Conclusions:**

In experimental CKD, BMAT expansion depends on CKD duration and does not appear to be driven by hypoleptinemia or hypercorticosteronemia, or by altered precursor cell differentiation during CKD onset.

## Introduction

Chronic kidney disease (CKD) is irreversible and results in the gradual loss of kidney function. Globally, more than ~800 million individuals world-wide present with CKD symptoms (1) and the mortality rate attributed to CKD is expected to reach ~2.2 million by 2040 (2). The progression of CKD leads to CKD–mineral bone disorder (CKD–MBD) which is characterized by disrupted mineral (calcium and phosphate) and hormonal (parathyroid hormone - PTH and fibroblast growth factor 23 - FGF-23) homeostasis, leading to cardiovascular disease and renal osteodystrophy (ROD) – the skeletal pathology component of the CKD-MBD syndrome (3).

The mechanism(s) responsible for the development of ROD are poorly understood but causative roles for hyperphosphatemia, hyperparathyroidism and elevated FGF-23 levels have been proposed (4). Indeed, increased systemic phosphate concentrations have been correlated with increased fracture risk in CKD patients (5). Also, FGF-23 is induced by hyperphosphatemia and this phosphaturic hormone can directly promote bone loss by inducing the expression of the Wnt/β-catenin signaling pathway inhibitor, Dickkopf-1 (6). Additionally, FGF-23 inhibits calcitriol production by the kidney, leading to decreased intestinal calcium absorption, secondary hyperparathyroidism and increased osteoclastic bone resorption (7). Clinical studies have reported that secondary hyperparathyroidism in CKD-MBD is positively correlated with fracture risk (8, 9).

Nevertheless, clinical and animal studies offer an alternative contributory explanation for the development of ROD. Bone marrow mesenchymal stromal cells (BMSC) can differentiate into various cell types, and osteoblast and adipocyte lineages are considered to have a ‘seesaw’ relationship with each requiring their own specific transcription factors (10). Therefore, the observation that bone marrow adipose tissue (BMAT) accumulation is inversely related to bone mass in CKD patients suggests a possible switch in stem cell commitment in CKD, resulting in a deficit of osteoblasts and thus bone formation (11). In experimental studies, the induction of CKD by nephrectomy or dietary adenine also results in BMAT accumulation and bone loss (12, 13).

In addition to peroxisome proliferator-activated receptor γ (PPARγ), a transcription factor essential for BMAT development, there are also a number of endocrine factors such as glucocorticoids, leptin and PTH that can influence BMAT accumulation (14, 15). Therefore, altered transcription factor expression and/or changes in the endocrine milieu that are characteristic of CKD may favor bone marrow adipogenesis rather than a commitment towards the osteoblast lineage. It is also possible that BMAT-secreted factors, such as leptin and adiponectin (16), can modulate osteoblast and osteoclast differentiation and cellular function (17). For example, BMAT secretes receptor activator of nuclear factor kappa beta ligand (RANKL) and C-X-C motif chemokine ligand 1 (CXCL1), which promote osteoclast differentiation and bone resorption (18, 19).

As the mechanisms responsible for BMAT accumulation in CKD and its relationship to bone loss is unclear, this study was undertaken to address this gap in our knowledge. Specifically, using a dietary adenine-induced CKD mouse model, we evaluated bone marrow adipocyte (BMAd) accumulation, bone structure and circulating adipokine concentrations during the early stages of CKD as well as quantifying BMSC, osteoblastic (OPC) and adipogenic (APC) precursor cell populations during CKD onset.

## Materials and methods

### Mice

All mouse studies were approved by the University of Edinburgh Animal Welfare and Ethical Review Board and were conducted under project licenses granted by the UK Home Office. Animal studies were conducted and are reported in line with the ARRIVE guidelines.

C57BL/6JCrl mice were obtained from Charles River Laboratories (Margate, Kent, UK). Seven-week-old male mice were acclimatized for 1-week after arrival into the animal facility prior to the start of the study. The animals were randomly assigned to control or CKD groups. The 8-week-old mice were subsequently fed their respective diet for up to 5 weeks: CKD mice received a casein-based diet containing 0.6% calcium, 0.9% phosphate, 1.5% Vitamin Mix, AIN-76A (containing vitamin D3), and 0.2% adenine (Envigo, Teklad Co. Ltd, Cat# TD.140290). Control mice received the same diet without adenine (Envigo, Cat# TD.138898) (20). Body weights were obtained from mice at the start of the experiment, and then at sacrifice after 1-, 3- and 5-weeks of induction.

For paired-feeding experiments, control and CKD mice were singly housed to facilitate the measurement of food intake by each mouse. This approach resulted in a rapid bodyweight loss (≥ 30% with ~ 1-week) in the adenine-fed mice, possibly due to enhanced stress of single housing. This study was therefore terminated for welfare reasons. Further attempts to prevent the rapid loss of body weight with increased bedding and acclimatization time were unsuccessful. Therefore, to complete the pair-feeding study, 6-week-old mice were acclimatized for 1-week in group housing, acclimatized in pairs for another 1-week and then randomly assigned to one of three groups: (1) *ad-lib* control diet, (2) *ad-lib* CKD diet and (3) paired-fed control diet for 3 weeks. Food intake of all cages was recorded daily. The amount of adenine diet consumed by both mice in each cage of group 2 was equal to the amount of control diet offered to its respective “paired” cage in group 3 the next day. In addition to their control or adenine-supplemented diet, mice were offered a gel pot (ClearH2O, USA, Cat# 7235022) for 2 days at the end of weeks 1 and 2. Over the 3-week duration of this study, this strategy was successful in preventing the adenine-fed mice breaching 30% body weight loss, which is the maximum allowed by our animal research license from the UK Home Office.

### Serum biochemistry

Control and CKD mice were sacrificed after 1-, 3-, and 5-weeks of induction and blood was collected by cardiac puncture under terminal anesthesia. Serum creatinine, blood urea nitrogen (BUN), were quantified using a biochemistry analyzer (Beckman Coulter AU480). Intact PTH (Immutopics International, USA, Cat# 60-2305), FGF-23 (Kainos Laboratories, Inc. Japan, Cat# CY4000), adiponectin (Sigma-Aldrich, UK Cat# A6354), leptin (R&D Systems, UK Cat# MOB00) and corticosterone (Enzo Life Sciences, UK, Cat# ADI-900-097) were measured by ELISA according to the manufacturers’ instructions.

### Micro-computed tomography

The left tibiae from control and CKD-mice, culled after 1-, 3-, or 5-weeks, were fixed in 10% formaldehyde for 24 hours prior to the evaluation of bone architecture and bone mineral density (BMD) by microCT (Skyscan 1172 X-ray microtomography, Bruker, Kontich, Belgium). Briefly, the bones were scanned with an isotropic voxel size of 5 μm (60 kV, 167 μA and 0.5 mm filter, 0.6° rotation angle) and the scans were reconstructed using the NRecon 1.7.3.0 program (Bruker) to remove artefacts, including beam-hardening and ring artefacts. CTAn software 1.15.4.0 (Skyscan) was used to visualize and evaluate bone histomorphometric parameters. Three-dimensional (3D) images were created using CTVol software (Skyscan).

To analyze the tibia architecture, each bone was aligned along its longitudinal axis and the trabecular volume of interest (VOI) in the proximal metaphysis was a 1000-µm section of the metaphysis, 250 µm subjacent to the growth plate (21). The following parameters were quantified: trabecular bone volume/tissue volume (BV/TV; %), trabecular thickness (Tb. Th; mm), trabecular number (Tb. N; 1/mm), trabecular separation (Tb. Sp; mm), and Tb. connectivity density (Tb. Conn Dn; 1/mm^3^). The cortical VOI was between 10 and 90% of the entire tibial length, as previously described (22). The proximal and distal 10% portions of tibial length were digitally cropped to exclude the epiphysis, growth plates and trabecular bone from the analysis. Cortical thickness (Th; mm), bone area (B.Ar; mm^2^), moment of inertia maximum (MMI max; mm^4^) and minimum (MMI min; mm^4^) and polar moment of inertia (*J*; mm^4^) were determined. Hydroxyapatite phantoms of known densities (0.25 and 0.75 g/cm^3^) were scanned and reconstructed under identical conditions as the experimental samples to allow the calculation of BMD. R studio was used to create the line graph of the cortical bone parameters along the tibia length (23).

### White adipose mass

Gonadal (gWAT) and inguinal (iWAT) fat depots were dissected from mice sacrificed after 1- and 3-weeks of induction. The weight of each peripheral fat depot was calculated as a % of body weight.

### Quantification of BMAT

After microCT scanning the tibiae were decalcified in 14% ethylenediaminetetraacetic acid pH 7.4 for 14 days at 4°C under agitation, with the solution changed every 2 - 3 days. The bones were stained with osmium tetroxide to evaluate BMAT accumulation within the bone marrow cavity (24). Briefly, the decalcified bones were washed and stored in 1.5 mL Eppendorf tubes containing 400 μl of Sorensens’ buffer (81 mM Na_2_HPO_4_·H2O, 19 mM KH_2_PO_4_, pH 7.4). Thereafter, 400 μl of 2% osmium tetroxide (Generon Ltd, Cat# 23310-10) was added to each tube to give a final concentration of 1% osmium tetroxide. After incubation for 48 hours at room temperature the bones were washed 2–3 times for a total of 1 hour with Sorensens’ buffer and stored in the same at 4°C.

The osmium-stained bones were re-scanned by microCT and BMAT volume was normalized to the size of the bone marrow cavity. BMAT volume was quantified in the whole tibia and also in 3 distinct anatomical regions: the top of the proximal bone to the growth plate (Proxi Epi); the growth plate to tibia/fibula junction (GP-T/F J), which contains regulated BMAT (rBMAT); and the tibia/fibula junction to the end of the distal bone (T/F J-End), which contains constitutive BMAT (cBMAT) (25). rBMAT is more labile and responds to metabolic challenges such as caloric restriction (CR), diabetes and ageing. R studio was used to create the line graph of BMAT accumulation along the tibia length.

### BMSC culture

To investigate the *ex-vivo* differentiation of osteoblast and adipocyte lineages, BMSCs were isolated from the tibiae, femora, humeri and ilia of CKD and control mice after 4-weeks on their respective diets. Briefly, BMSCs were obtained by centrifugation at 13,000x g at room temperature for 1 minute and the cell clump was gently broken-up using a 1-ml syringe with 25 G needle. Bone marrow cells were pooled separately from each group (control and CKD mice) and expanded in minimum essential medium alpha (αMEM, Life Technologies, Cat# 22561021), 10% fetal bovine serum (FBS, Life Technologies, Cat# 10500064), and 1% penicillin/streptomycin (referred to as BMSC medium) for 48 hours. For adipocyte and osteoblast differentiation, BMSCs were seeded in 6-well plates at a density of 5 × 10^5^ cells/well and maintained in BMSC media until the cells reached confluency (day 0). For osteoblast differentiation, confluent BMSCs were cultured in osteoblast differentiation medium (BMSC media containing 50 μg/mL ascorbic acid and 8 mM β-glycerophosphate) for 21 days, with medium replaced in every 2–3 days. For adipocyte differentiation, confluent BMSCs were cultured in adipocyte induction medium, which consisted of adipocyte-basal medium (Dulbecco Modified Eagle Medium, high-glucose, supplemented with 10% FBS, 1% penicillin/streptomycin, 1 μM rosiglitazone and 2 μM insulin) supplemented with 0.5 mM 3-isobutyl-1-methylxanthine and 1 μM dexamethasone. Medium was changed on day 2. On day 4, adipocyte induction medium was switched to adipocyte-basal medium and the cells were cultured for another 5 days, with medium changes every other day (26).

### Alkaline phosphatase activity and alizarin red and oil red O staining.

Alkaline phosphatase (ALP) activity and Alizarin Red staining of cultured BMSCs from control or CKD mice were determined on day 21 of osteogenic differentiation. Cells were lysed with 0.2% triton X-100 to release intracellular ALP. The ALP activity assay (Sigma-Aldrich, Cat# MAK447) used p-nitrophenyl phosphate as substrate and the production of p-nitrophenol was measured at an absorbance of 405 nm. The mineralized matrix in each well was stained with a 2% solution of Alizarin Red at pH 4.2 at room temperature for 5 min. After rinsing several times with water, the bound stain was solubilized in 10% cetylpyridinium chloride and the optical density of the resultant eluted solution was measured by spectrophotometry at 570 nm (27). On day 9 of adipogenic differentiation, adipocytes were stained with Oil Red O. Briefly, adipocytes were fixed in 10% neutral buffer formalin for 30 min at room temperature. After rinsing with water, 60% isopropanol was added for 5 mins at room temperature. The adipocytes were stained with Oil Red O solution for 5 min, washed with water and the bound stain solubilized in isopropanol. The optical density of the resultant eluted solution was measured by spectrophotometry at 490 nm. ALP activity, Alizarin Red staining and Oil Red O staining were normalized to total cellular protein, as determined by the Quick start Bradford protein assay (Bio-Rad, UK, Cat# 5000201) according to the manufacturers’ instructions.

### Flow cytometry

Control and adenine-fed mice were sacrificed after 1- or 2- weeks on their respective diets. Bone marrow from tibiae and femora was isolated by centrifugation as described above and suspended in 1 ml of cold cell-suspension buffer (sterile PBS with 2% FBS and 100 U/ml penicillin/streptomycin). Hematopoietic cells were depleted by the addition of ammonium-chloride-potassium lysis buffer for 5 minutes. Cells were resuspended in cell-suspension buffer, followed by blocking of non-specific protein binding sites using 2% rabbit serum in PBS for 30 minutes. Thereafter, the cells were incubated for 1 hour with the following fluorochrome-conjugated monoclonal antibodies (all from Thermo Fisher Scientific, UK): anti-CD45 (Cat# 11-0451-82), CD31 (Cat# 11-0311-82), Sca1 (Cat# 17-5981-82), Pa (Cat# 12-1401-81) and CD24 (Cat# 47-0242-82). Zombie Violet (BioLegend, USA, Cat# 423113) was used as a dead cell marker. Fluorescence minus one (FMO) was applied for each antibody as a negative control to inform gating boundaries. Flow cytometry was performed on a BD LRSFortessa™ and cells were identified using FlowJo software according to the following surface markers: BMSC, CD31-CD45-Sca1+Pa+CD24+; APC, CD31-CD45-Sca1+Pa+CD24-, and OPC, CD31-CD45-Sca1-Pa+ (28).

### Quantitative polymerase chain reaction

Isolated bone and bone marrow were snap frozen in liquid nitrogen and stored at -80^0^C. Cells were thawed, lysed in 1 ml of Qiazol (Qiagen, Manchester, UK, Cat# 79306) and homogenized with a Rotor-Stator Homogenizer (Ultra-Turrax T10). RNA from both tissues was extracted using a Qiagen RNeasy Mini kit (Qiagen, Cat# 74106) and quantified by nanodrop spectrophotometry (Thermo Fisher Scientific, Loughborough, UK). RNA quality was evaluated by the 260/280 nm ratio. All RNA was diluted to the same concentration and reverse transcribed to cDNA using SuperScript II (Thermo Fisher Scientific, Cat# 18064022). To evaluate gene expression, cDNA was mixed with PrimerDesign PrecisionPlus Master Mix and premixed SYBR Green (PrimerDesign, Chandler’s Ford, UK, Cat# PB20.11-05) and assessed by a Stratagene Mx3000P real-time qPCR system (Agilent Technologies, Cheadle, UK). Target gene expression was normalized to a housekeeping gene (*Ppia*) and analyzed using the ΔΔCt method. Oligonucleotide primers (**Supplementary Table S1**) were obtained from Sigma-Aldrich.

### Statistics

Data are presented as mean ± standard error of the mean (S.E.M). Each dot in all bar graphs represents 1 mouse sample in each study. Statistical analysis was performed using a two-way analysis of variance (ANOVA) to determine the effect of time and CKD status on bone and BMAT alterations. Correlations between individual parameters were performed using Spearman correlation. Statistical analysis was implemented using GraphPad Prism software (GraphPad Software, Inc., USA) and R studio (for the whole cortical bone and BMAT analysis) and statistical significance was shown as; * p<0.05; ** p<0.01 and *** p<0.001.

## Results

### Validation of disease progression in the CKD mouse model

**Figure 1:**
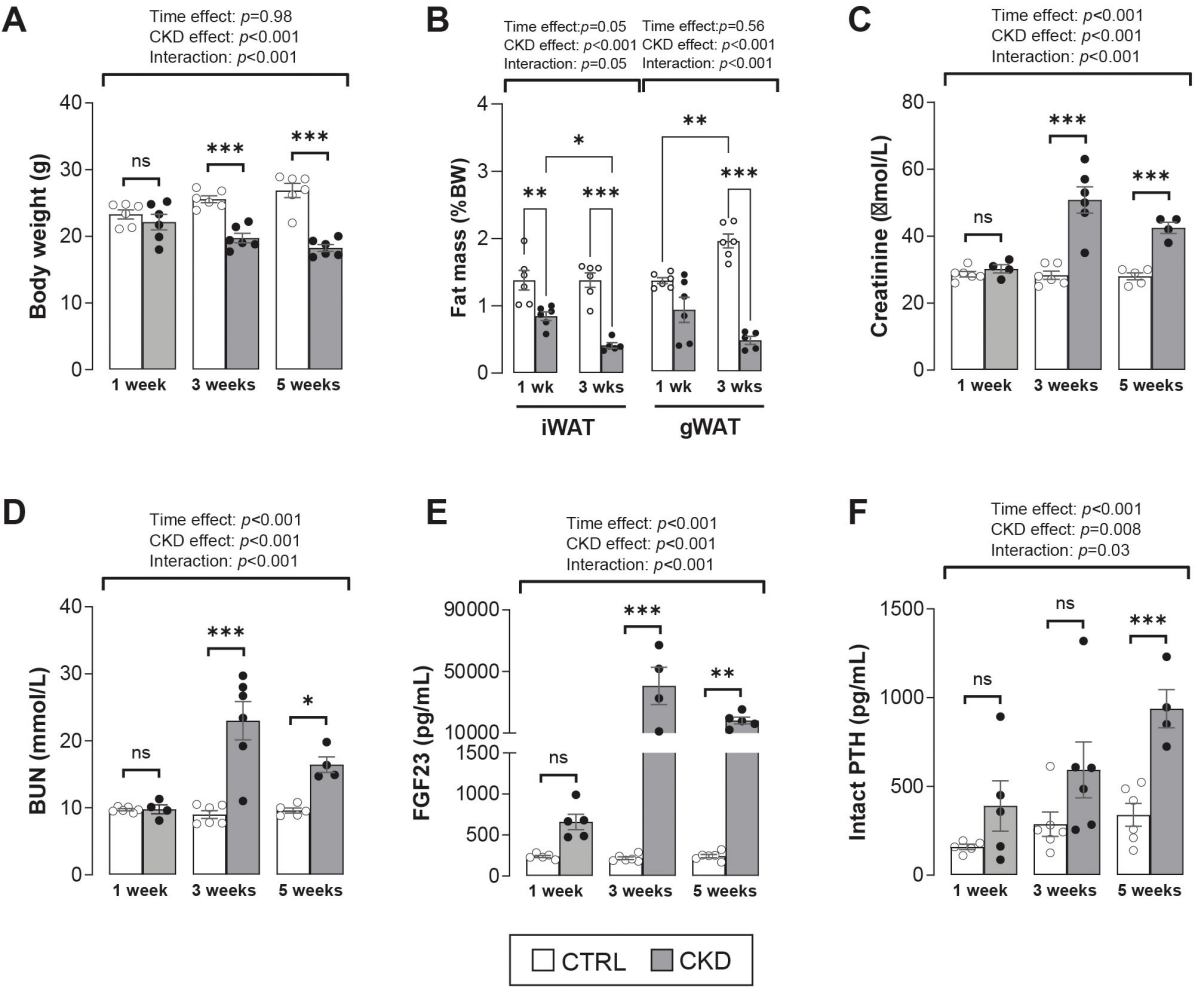
Validation of CKD development in adenine-treated mice. **(A)** Body weight of CKD mice was decreased after 3- and 5- weeks’ induction. **(B)** Inguinal (iWAT) and gonadal (gWAT) white adipose tissue of CKD mice was decreased after 1- and 3-weeks’ induction. **(C–E)** Serum levels of creatinine, blood urea nitrogen (BUN), and fibroblast growth factor 23 (FGF23) were increased from 3-weeks post-induction, whereas **(F)** parathyroid hormone (PTH) serum levels were increase after 5-weeks’ induction. The data are presented as mean ± SEM, with each dot representing an individual mouse. ns, not significant, *P < 0.05, **P < 0.01, ***P < 0.001.

The temporal development of the CKD phenotype in mice fed an adenine-supplemented diet was verified. The body weight of CKD mice decreased from 3-weeks’ induction when compared to age-matched control mice (**Figure 1A**). This coincided with a decrease in fat mass in the gWAT and iWAT depots (**Figure 1B**). After 1-week of induction, serum creatinine, BUN, FGF-23 and PTH were similar in age-matched control and adenine-fed mice, but after 3- or 5- weeks’ induction these analytes were increased in the CKD mice (**Figures 1C–F**). These data confirm the temporal development of CKD in the experimental mice and suggest that in this model “CKD onset” and the “early stages of CKD” occur after 1 – 2, and 3–5 weeks of induction, respectively (20).

### Cortical and trabecular bone structure is impaired in CKD mice

**Figure 2:**
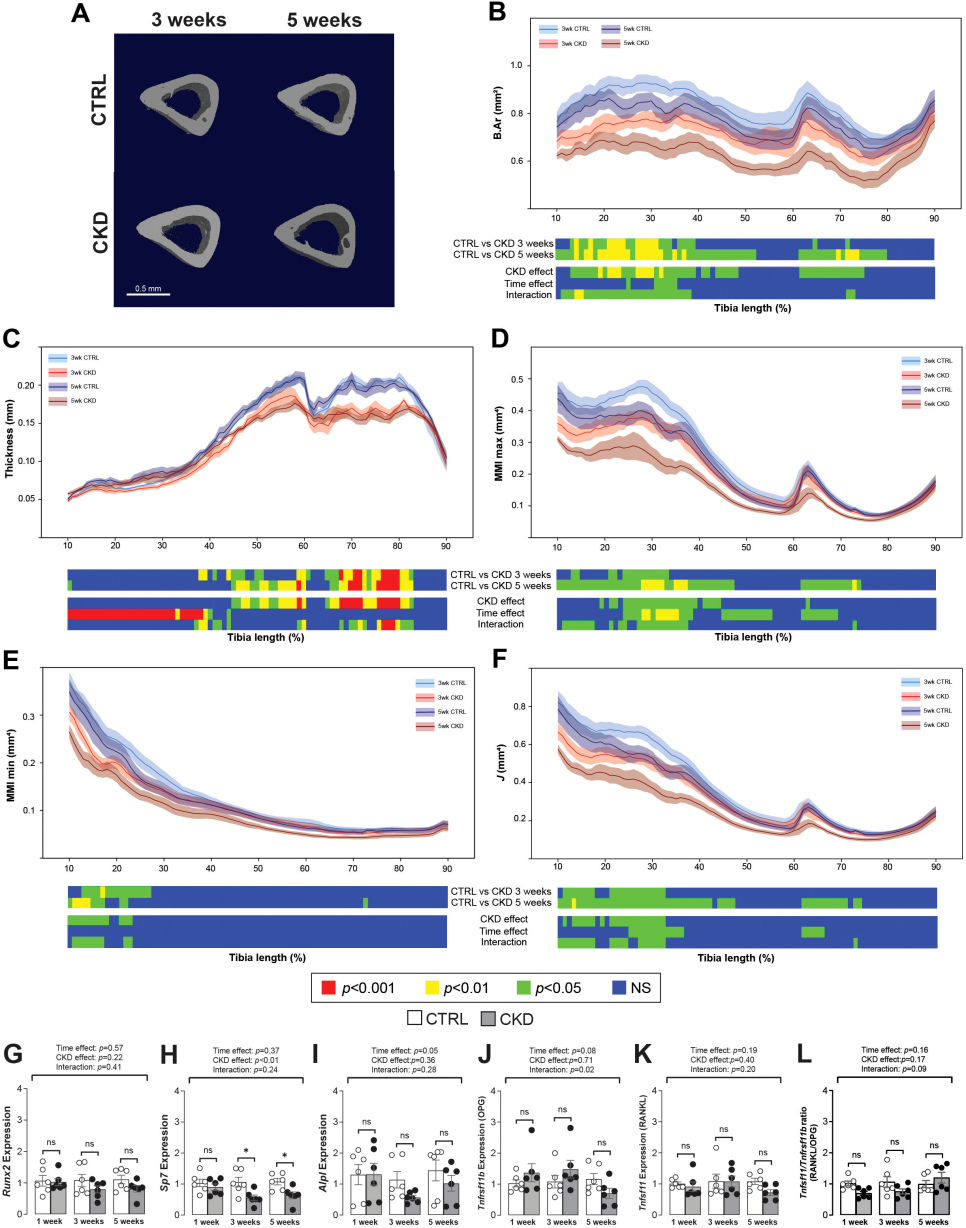
Micro-CT analysis and gene expression of tibial cortical bone. **(A)** 3-dimentional images of cortical bone from CTRL and CKD mice after 3- and 5-weeks’ induction. Quantification of whole bone analyses of cortical bone between 10 and 90% of total tibial length, excluding proximal and distal metaphyseal bone, of CTRL and CKD tibia after 3- and 5-weeks’ induction. **(B)** bone area (B.Ar), **(C)** thickness, **(D)** maximum moment of inertia (MMI max), **(E)** minimum moment of inertia (MMI min), and **(F)** polar moment of inertia **(J)** were all reduced in various regions of the tibia following CKD induction. For clarity, data for 1-week CTRL vs CKD are shown in **Supplementary Figure S1**. Data are presented as mean ± SEM, n = 5–6 bones per group. The expression of **(H)** *Sp7* was decreased after 3- and 5-weeks’ induction whereas the expression of **(G)** *Runx2*, **(I)** *Alpl*, **(J)** *Tnfrsf11b*, and **(K)** *Tnfsf11* as well as the **(L)** *Tnfsf11/Tnfrsf11b* ratio was similar in the tibia from CTRL and CKD mice at all sampling points. The data are presented as mean ± SEM, with each dot representing an individual mouse. ns, not significant, *P < 0.05.

We next investigated how CKD duration impacts cortical and trabecular bone structure. In the CKD mice, cortical bone area was decreased in the proximal tibia after 3-weeks’ induction but after 5-weeks the bone area of both the proximal and distal bone was reduced (**Figure 2A** and **2B**). Cortical thinning was most evident in the distal tibia of CKD mice (**Figure 2C**) whereas MMI max and min as well as the polar moment of inertia (*J*) were decreased particularly in the proximal tibia of the CKD mice (**Figures 2D–F**). No differences for CKD vs control were noted after 1-week of induction (**Supplementary Figure S1**).

Changes to the trabecular compartment were observed only after 5-weeks’ induction. When compared to age-matched controls, bone volume fraction and trabecular BMD, thickness, number and connectivity were all decreased in CKD mice after 5-weeks’ induction (**Supplementary Figure S2**).

### Osterix expression in cortical bone is decreased in CKD mice

In cortical bone, the expression of *Runx2* was unaltered whereas *Sp7* was decreased after 3- and 5-weeks’ induction (**Figures 2G, H**). *Alpl*, *Tnfrsf11b* and *Tnfsf11* were similarly expressed in control and CKD cortical bone at all stages of disease progression, with only a weak time-dependent variation observed for *Alpl* (**Figures 2I–K**). The *Tnfsf11/Tnfrsf11b* ratio was similar in control and CKD cortical bone after 1-, 3- and 5-weeks’ induction (**Figure 2L**).

### CKD mice have increased BMAT accumulation

**Figure 3:**
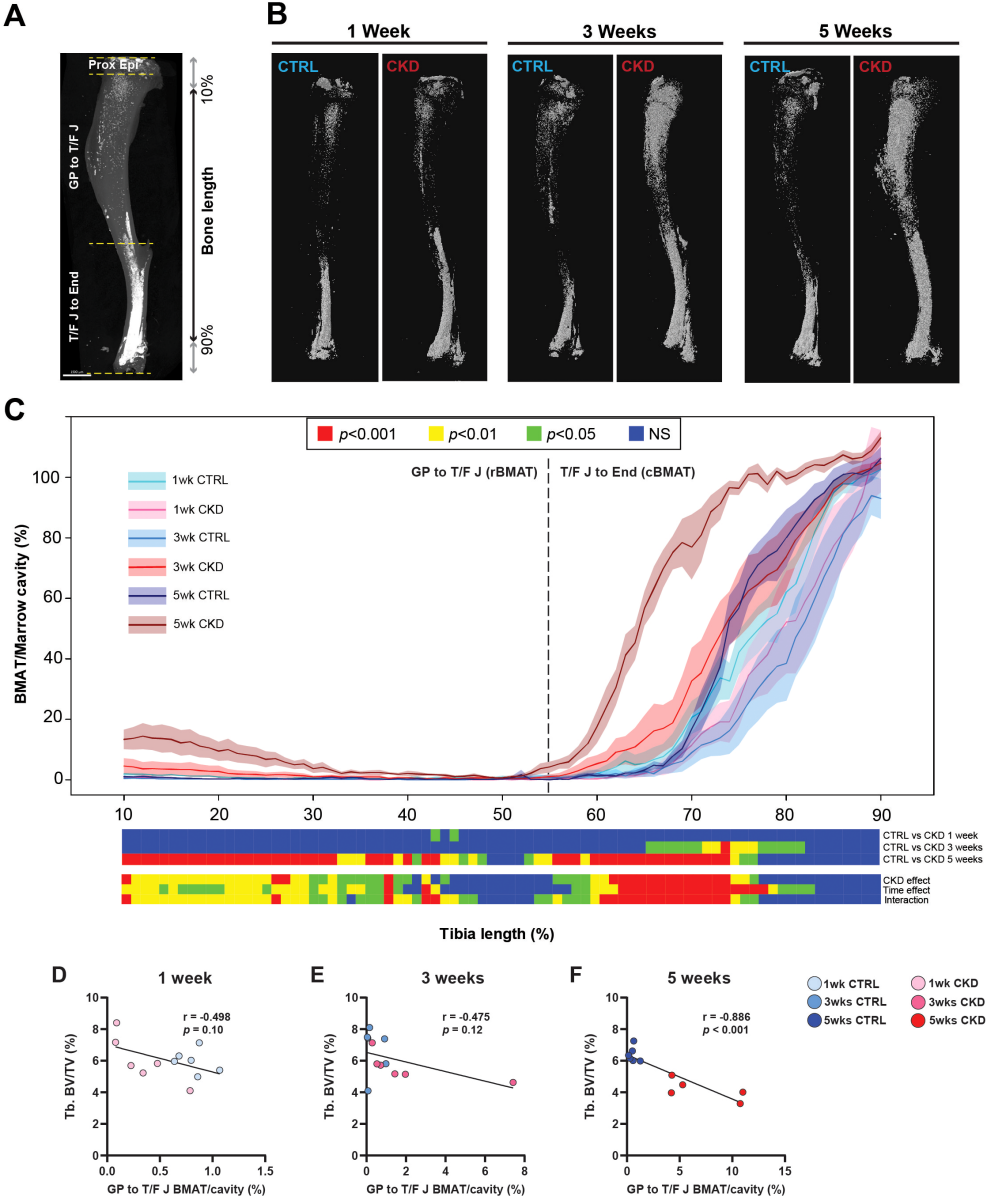
BMAT distribution along the tibial bone shaft and its correlation with trabecular bone. Visualization and quantification of bone marrow adipose tissue (BMAT) within the marrow cavity of CTRL and CKD tibia after 1-, 3- and 5-weeks’ induction. **(A)** distribution of osmium stained BMAT within the different anatomical regions of the tibia: the top of the proximal bone to the growth plate (Proxi Epi); the growth plate to tibia/fibula junction (GP-T/F J), which contains regulated BMAT (rBMAT); and the tibia/fibula junction to the end of distal bone (T/F J-End), which contains constitutive BMAT (cBMAT). **(B)** distribution and **(C)** quantification of rBMAT and cBMAT in CTRL and CKD mice. cBMAT was increased in CKD mice at 3- and 5-weeks after induction whereas an increase in rBMAT in CKD mice was only noted after 5-weeks’ induction. **(D–F)**. The dashed line represents the junction between rBMAT and cBMAT. Data are presented as mean ± SEM, n = 5–6 bones per group. **(D–F)** Correlation analysis between trabecular bone volume fraction (Tb.BV/TV) and rBMAT indicated a negative correlation after 5-weeks’ induction but not after 1- or 3-weeks. Each dot represents an individual mouse.

To establish whether altered bone structure in the CKD mice was related to changes in BMAT accumulation and distribution, we next quantified BMAT along the tibia length as visualized by microCT images of osmium-stained adipose tissue (**Figures 3A, B**). After 5-weeks’ induction, rBMAT of CKD mice was increased in the proximal tibia (~10 - 48% region of the tibia length). Similarly, cBMAT of CKD mice was increased in the ~65 - 82% and ~52 - 78% regions of the tibial length after 3- and 5- weeks’ induction, respectively (**Figures 3C** and **S3**). We next determined if BMAT accumulation within the trabecular compartment was at the expense of juxtaposed bone formation. No differences in trabecular bone structure were noted in CKD mice after 3-weeks’ induction (**Supplementary Figure S2**); a time point when there was increased constitutive and total BMAT and a trend for increased rBMAT (**Figures 3C** and **S3**). Correlation analysis revealed an inverse relationship between rBMAT and trabecular bone volume fraction after 5-weeks’ induction but not at the earlier time points (**Figures 3D–F**). These results indicated that rBMAT accumulation tended to occur prior to trabecular bone loss and that there was a negative correlation between rBMAT accumulation and trabecular bone loss during the early stages of CKD.

### Hormonal changes and their correlations with BMAT

**Figure 4:**
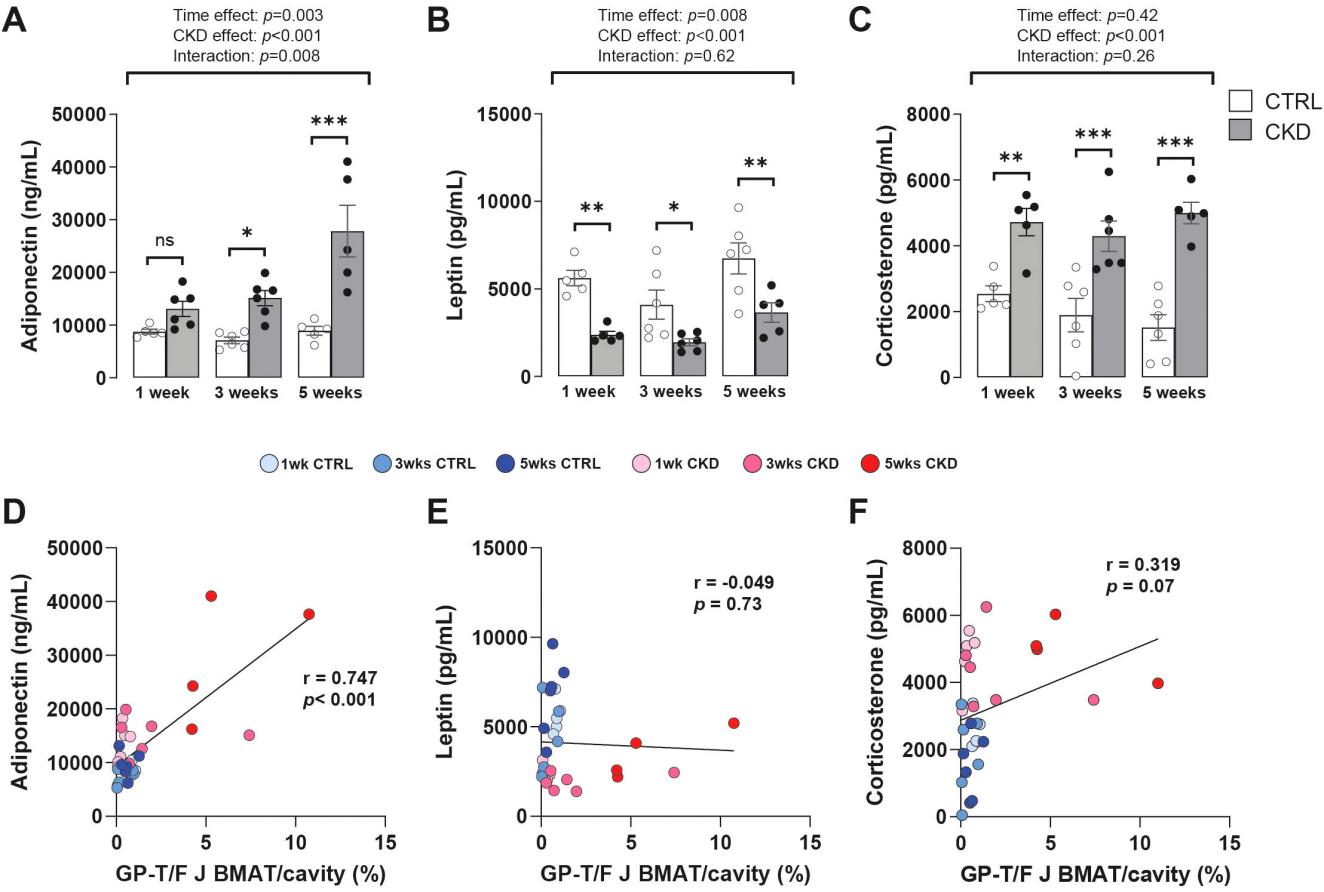
Hormonal changes and their correlation with rBMAT accumulation. Serum levels of **(A)** adiponectin **(C)** and corticosterone were increased in CKD mice following 3- and 1- weeks’ induction, respectively, whereas **(B)** leptin levels were decreased at all sampling points. Data are presented as mean ± SEM, with each dot representing an individual mouse. ns, not significant, *P < 0.05, **P < 0.01, ***P < 0.001. **(D)** Circulating adiponectin exhibited a positive correlation with rBMAT (GP-T/F BMAT) but no such correlation was observed with **(E)** leptin or **(F)** corticosterone.

During calorie restriction (CR), changes in circulating adiponectin, leptin, and glucocorticoids have been proposed as consequences or causes of BMAT expansion (19); hence, we tested these relationships in our CKD model. Plasma levels of adiponectin in CKD mice increased in a time-dependent manner, being higher in CKD mice than in age-matched control mice after 3- and 5- weeks’ induction (**Figure 4A**). In contrast, the concentrations of plasma leptin or corticosterone in CKD mice were lower or higher, respectively, than in control mice at all ages studied (**Figures 4B, C**).

These results suggest that increased adiponectin is associated with other duration-dependent effects of CKD, including on BMAT, whereas the changes in leptin and corticosterone precede these other CKD effects. To test these relationships, the correlation between rBMAT accumulation and plasma levels of adiponectin, leptin and corticosteroids in control and CKD mice was determined. This revealed that circulating adiponectin was positively correlated with rBMAT accumulation whereas both circulating leptin and corticosterone were not (**Figures 4D–F**).

### BMAT accumulation in CKD is not due to caloric restriction

The altered hormonal profile of the CKD mice was similar to that observed in CR. Therefore, to determine whether increased BMAT in the CKD mice might be caused by CR rather than CKD *per se*, a pair-feeding experiment was carried out. Mice fed the CKD diet *ad-lib* consumed less than those fed the control diet *ad-lib* and this difference in food intake was reflected in their body weights (**Supplementary Figures S4A, B**). To prevent excessive, pathological weight loss, all mice had *ad-lib* access to a supplementary gel pot diet for 48 h periods at the ends of week 1 and 2 (**Supplementary Figures S4A, B**, green shading). The CKD mice lost more body weight than the pair-fed mice despite a similar caloric intake over the 3 weeks of the study, suggesting that CKD was having effects beyond those explainable by CR alone (**Supplementary Figures S4A, C**). Consistent with this, BMAT accumulation was similar between control and pair-fed mice but was greater in CKD mice than in either of these groups (**Supplementary Figure S4D**). These results suggest that the accumulation of BMAT was a consequence of CKD progression and not of CR *per se*.

### Gene expression in bone marrow tissue is altered in CKD mice

**Figure 5:**
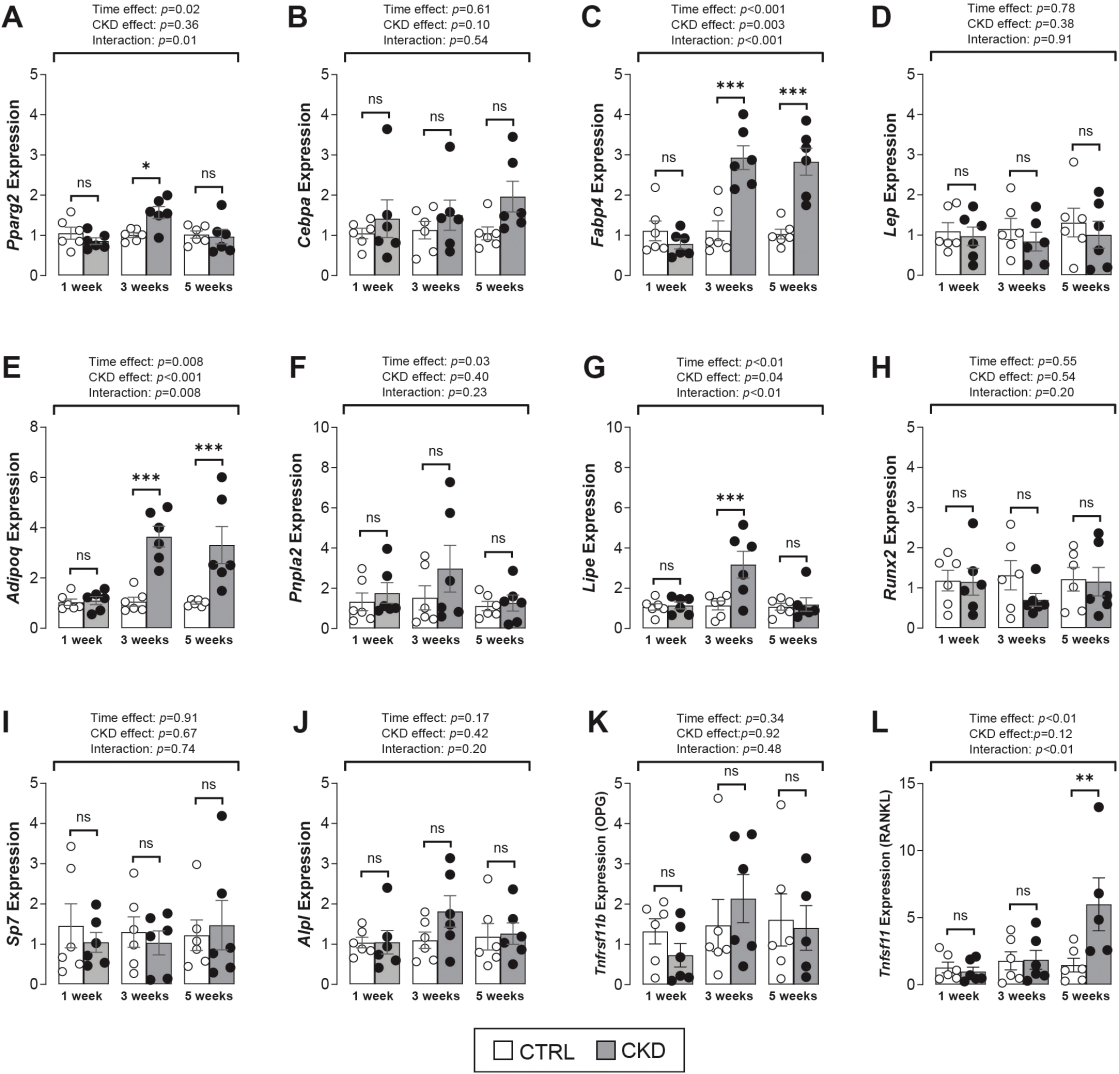
Adipogenic and osteogenic gene expression in bone marrow tissue. **(A–E)** The expression of the adipogenic transcription factor genes, *Pparg2* and *Cebpa* were similar in CTRL and CKD samples whereas the mature adipocyte marker genes, *Fabp4* and *Adipoq* but not *Lep* were increased in CKD marrow after 3- and 5-weeks’ induction. There was a tendency for an increase in the expression of the lipolytic genes, **(F)** *Pnpla2* and **(G)** *Lipe* after 3-weeks’ CKD induction but this difference only reached significance for *Lipe* expression. The expression of the osteogenic transcription factor genes **(H)** *Runx2*, **(I)** *Sp7*, **(J)** *Alpl* and **(K)** *Tnfrsf11b* were similar in CKD and CTRL mice while **(L)** *Tnfsf11* expression was increased in CKD mice after 5-weeks’ induction. Data are presented as mean ± SEM, with each dot representing an individual mouse. ns, not significant, *P < 0.05, **P < 0.01, ***P < 0.001.

To investigate if bone marrow progenitor fate is altered in CKD mice, we next assessed the expression of key transcription factors and phenotype-specific genes in bone marrow tissue from control and CKD mice. For adipogenic markers, there was no effect of CKD on *Pparg2, Cebpa* or *Lep* expression whereas two other markers of mature adipocytes, *Fabp4* and *Adipoq*, were significantly increased in the bone marrow of CKD mice at 3- and 5-weeks of disease progression (**Figures 5A–E**). The expression of the lipolytic genes, *Pnpla2* and *Lipe* was similar in control and CKD bone marrow but tended to be higher in CKD mice after 3-weeks’ induction, with this difference reaching significance for *Lipe* expression (**Figures 5F, G**). For osteogenic markers, the expression of *Runx2*, *Sp7*, *Alpl* and *Tnfrsf11b* were similar in bone marrow of CKD and control mice, whereas *Tnfsf11* expression was higher in CKD mice after 5-weeks’ induction when compared to age-matched controls (**Figures 5H–L**).

### The number of adipocyte and osteoblast progenitor cells are unchanged during CKD onset

**Figure 6:**
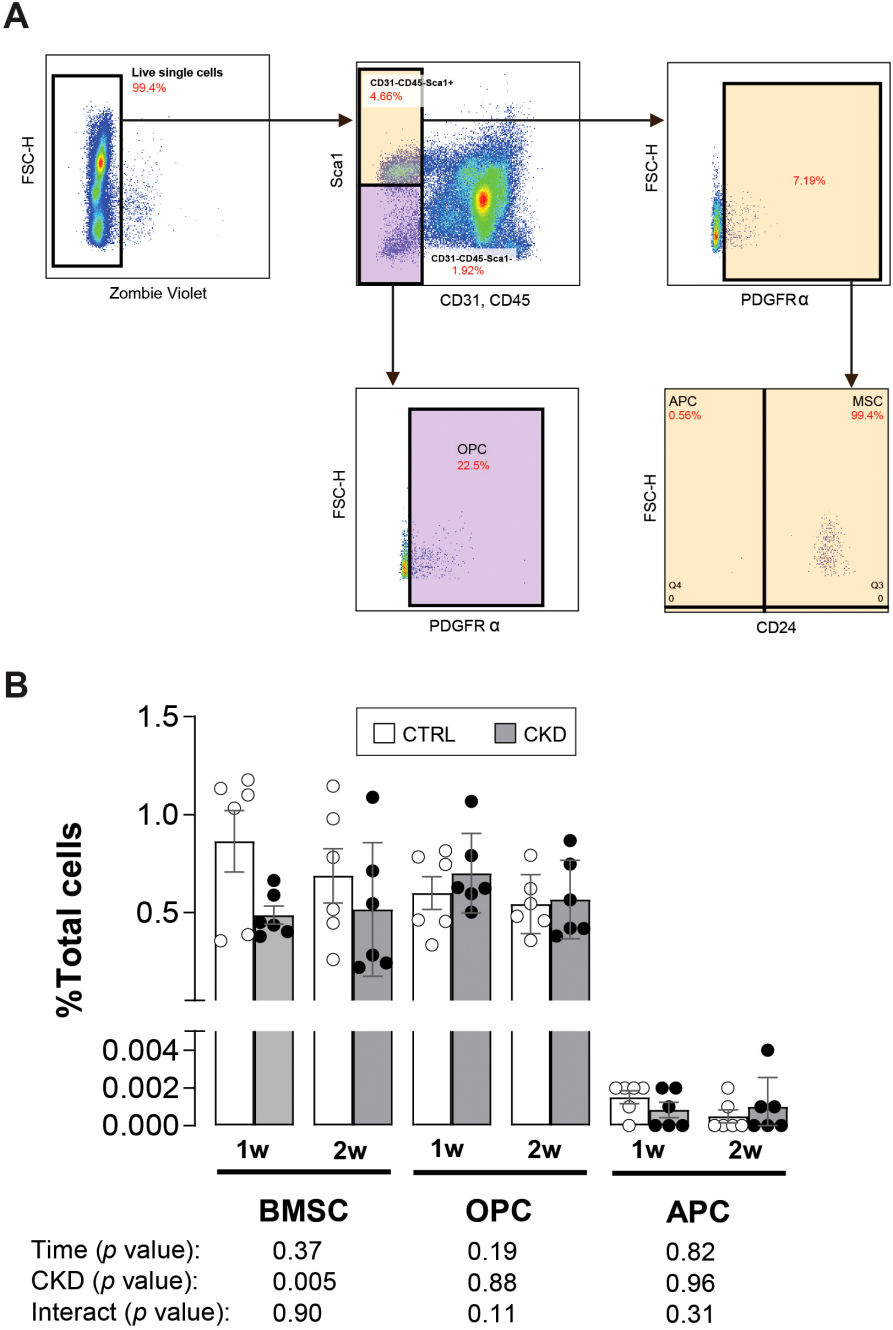
APC and OPC populations in CKD and CTRL mice. **(A)** The populations of bone marrow mesenchymal stromal cells (BMSCs), osteoblastic (OPC) and adipogenic (APC) precursor cells from the bone marrow of CTRL and CKD after 1- and 2-weeks induction were gated by CD45-CD31-Sca1+CD24+, CD45-CD31-Sca1+CD24- and CD45-CD31-Sca1-Pa+, respectively. **(B)** The BMSC population as a percentage of all live cells (total cells) was reduced in the CKD mice whereas the OPC and APC populations as a percentage of total cells was unchanged. Data represented as mean ± SEM, with each dot representing an individual mouse. Sca1; stem cell antigen 1, PDGFRα; platelet-derived growth factor-α.

The observation that the expression of *Fabp4* and *Adipoq* was higher in CKD bone marrow tissue (**Figure 5**) and that BMAT in CKD mice started to accumulate from 3-weeks of induction (**Figure 3**) suggests that in CKD there may be a switch, prior to the 3-week time point, in BMSC commitment towards the adipocyte rather than the osteoblast phenotype. To address this possibility, we used flow cytometry to analyze bone marrow from control and CKD mice, 1- and 2- weeks after induction, to quantify the number of BMSCs, OPCs and APCs (**Figure 6A**). The BMSC population in CKD bone marrow was reduced from control levels after 1- and 2- weeks’ induction whereas the proportions of OPCs and APCs were similar in bone marrow from CKD and control mice at both time points studied (**Figure 6B**). These results indicate that BMAT accumulation in CKD mice is unlikely to be driven by a prior change in BMSC commitment but it is possible that 1 or 2-weeks of dietary adenine intake was insufficient to program such changes in BMSC lineage commitment.

### Expression of *Adipoq* and adipocyte transcription factors is increased in BMSCs from CKD mice

**Figure 7:**
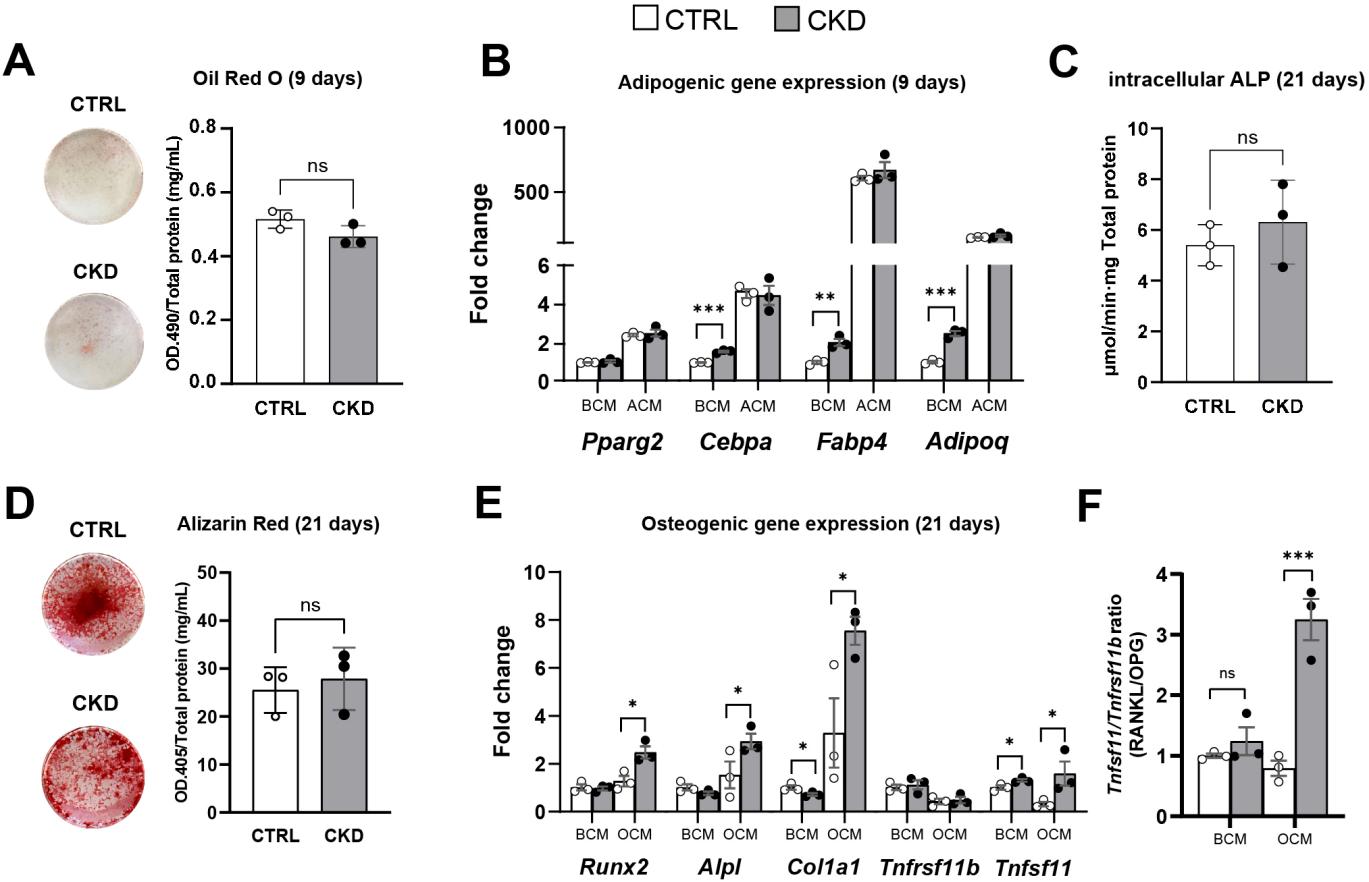
Adipocyte and osteoblast differentiation of isolated primary BMSC from control and CKD mice. Primary BMSC from CTRL and CKD mice were cultured in BMSC cell culture media (BCM), adipogenic cell culture media (ACM) for 9 days and osteogenic cell culture media (OCM) for 21 days. **(A)** After 9 days in ACM, oil red O staining of adipocytes was similar in CTRL and CKD cultures. **(B)** Expression of the adipogenic genes, *Cebpa*, *Fabp4* and *Adipoq* were increased in the BMSC from CKD mice cultured in BCM. In contrast, while ACM induced *Pparg2*, *Cebpa*, *Fabp4* and *Adipoq* gene expression in BMSC from both CTRL and CKD mice the expression levels were similar in CTRL and CKD cultures. In primary BMSC cultured in OCM for 21 days, both **(C)** intracellular ALP activity and **(D)** alizarin red staining was similar in CTRL and CKD cultures. **(E)** The expression of osteogenic genes by BMSC cultured in BCM revealed that *Col1a1* was decreased whereas *Tnfsf11* was increased in CKD samples. When primary BMSC were cultured in OCM, osteogenic genes *Runx2, Alpl, Col1a1* and *Tnfsf11* were increased in CKD samples. **(F)** The *Tnfsf11/Tnfrsf11* ratio was similar in CTRL and CKD BMSC cultured in BCM but increased when cultured in OCM. Data are presented as mean ± SEM with each dot representing an individual culture. ns, not significant, *P < 0.05, **P < 0.01, ***P < 0.001.

To further explore the effects of CKD on BMSC lineage commitment, we next determined whether disease progression directly affects the differentiation of BMSCs to mature adipocytes and osteoblasts rather than simply their commitment to APCs or OPCs, as studied above (**Figure 6**). We isolated primary BMSCs from mice that had been on control or CKD diets for 4-weeks, when CKD-induced BMAT accumulation was established (**Figure 3**). After nine days of adipogenic induction, BMSCs from control and CKD mice had similar oil red O staining and expression of the adipogenic genes *Cebpa*, *Fabp4*, *Adipoq* and *Pparg2* (**Figures 7A, B**). In contrast, when cultured in BMSC medium the expression of *Cebpa*, *Fabp4* and *Adipoq* but not *Pparg2* was upregulated in CKD cells compared to control cells (**Figure 7B**). This incongruity is likely explained by the overpowering capacity of adipogenic medium to induce adipocyte gene expression and dwarf any programming effects of CKD.

For osteoblast differentiation, BMSC from control and CKD mice had similar ALP activity and Alizarin Red staining after 21 days in osteogenic medium (**Figures 7C, D**) but the expression of *Runx2*, *Alpl*, *Col1a1* and *Tnfsf11* was upregulated in CKD cells compared to control cells (**Figure 7E**). In contrast, the expression of the osteogenic genes by control and CKD cells cultured in BMSC medium was similar (**Figure 7E**). The *Tnfsf11/Tnfrsf11* gene ratio was similar in control and CKD BMSC cultured in BCM but increased when cultured in OCM (**Figure 7F**). These discrepant gene expression data may be a result of differences in responsiveness between control and CKD cells to osteogenic medium. It is of course possible that increased sample number and longer culture periods may have revealed further differences in gene expression and in oil red O and alizarin red staining.

## Discussion

It is widely recognized that CKD can lead to bone loss, thereby increasing the risk of osteoporosis and fractures (29). Bone loss and increased serum bone turnover markers have also been noted in a variety of animal models of CKD-MBD and while the precise mechanisms responsible for the development of ROD remains unclear, increased bone resorption due to chronically high PTH levels is regarded as central to its etiology (8, 9, 20). However, other mechanisms such as uremic-toxin-induced mitochondrial abnormalities are likely to also be involved, as increased hip and vertebral fractures have been reported in patients with low circulating PTH (30, 31).

Our finding of increased BMAT in this mouse model of CKD is consistent with previous observations in other animal models (12, 13) and in humans with this disease (32, 33). There are several mechanisms through which increased BMAT may directly contribute to bone loss and fracture risk in CKD. One possibility is that a switch in the commitment of BMSC from the osteoblast to the adipocyte lineage may offer an additional/alternative explanation for the development of ROD. A change in mesenchymal cell fate may also explain the higher marrow fat content in patients with osteopenia/osteoporosis (34, 35), ageing (36, 37), obesity (38) and CR (26). An inverse relationship between bone mass and BMAT is observed in the CKD mice of the present study and a CKD-specific determinant of BMAd accumulation could be PTH. When administered intermittently, PTH is anabolic to the skeleton and reduces BMAT in mice and in male and female osteoporotic patients (18). However, the influence of chronically high levels of PTH, as observed in CKD patients and CKD animal models, on BMAT accumulation is unknown. In CKD patients, BMAT accumulation is positively associated with disease severity. For example, Woods et al. showed that BMAT is greater in individuals with an estimated glomerular filtration rate (eGFR) of ≤45 mL/min compared to those with an eGFR of >60mL/min (33) and Borelli and colleagues reported that BMAT accumulation in CKD patients is negatively correlated to eGFR and volumetric BMD (11). Clinical studies in non-CKD patients have also shown that BMAT accumulation correlates with vertebral fracture risk, and in ageing adults this risk is independent of BMD, suggesting that factors released from BMAds such as RANKL may contribute to poor bone quality (39, 40). Moreover, variants in the RANKL gene (*TNFSF11)* are associated with altered bone marrow adiposity in humans (37), further highlighting the links between BMAT and bone remodeling. Whether BMAT is associated with fracture incidence in CKD patients is yet to be determined.

To establish whether BMAT accumulation and bone loss noted in CKD is a result of a switch in BMSC differentiation to favor adipogenesis at the expense of osteoblast formation, we first established a longitudinal mouse model of CKD-BMD to record the temporal relationship between BMAT accumulation and bone mass during disease progression. Our findings reveal an inverse relationship between trabecular bone mass and rBMAT accumulation during the progression of CKD, which is consistent with adult and pediatric CKD data (11, 41).

However, our data also suggest that BMAT accumulation precedes bone loss in CKD. We show that body weight loss and serum CKD markers first become apparent after 3-weeks in CKD mice, by which time there is an increase in total BMAT accumulation and *Fabp4* and *Adipoq* expression in CKD bone marrow tissue but no change in trabecular BMD, BV/TV, number, thickness or connectivity. These data confirm the complex relationship between BMAT accumulation and bone loss. While an increase in adipogenesis in bone marrow stromal cells and a reduction in osteogenic differentiation occurs in both CR and ageing (26, 42), other studies have reported BMAT accumulation *per se* does not lead to bone loss (43, 44). Also, studies with rabbits have disclosed that during CR, bone loss can occur independently of BMAT accumulation (19). Furthermore, recent studies have identified the presence of two distinct BMSC populations and questioned the mutually exclusive commitment of BMSC to the adipogenic or osteogenic lineages (45). Therefore, more definitive experiments are required which could involve inducible Cre lines that exclusively label pre-commitment cellular population *e.g.* Cxcl12-CreE (46). This approach would allow the visualization of stromal cell populations *in vivo*.

From our data it is possible that additional factors beyond lineage differentiation may contribute to BMAT accumulation in CKD. Nevertheless, we reveal that the proportion of BMSCs decreases during CKD onset and, while this suggests a potential impairment of BMSC proliferation and/or survival, further studies are required to confirm this. Also, the differentiation of primary BMSCs from 4-week-induced CKD mice towards the adipocyte and osteoblast phenotype may be insightful. The increased expression of adipogenic genes in CKD BMSC cultured in BMSC medium suggests an inherent predisposition towards adipogenesis. This may be attributed to long-term exposure to an altered systemic milieu in the CKD mice that may prime BMSCs for adipogenic differentiation. The preferential upregulation of osteogenic genes in CKD BMSC cultured in osteogenic media suggests a potentially heightened, yet potentially dysfunctional, osteogenic response.

Our finding of higher serum levels of adiponectin in the CKD mice mirrors human data where, in CKD individuals, it may act as a biomarker for renal dysfunction (47). The correlation between serum adiponectin and BMAT volume supports the possibility that the accumulated BMAT is the source of the increased adiponectin in the CKD mice, as noted in mouse models of CR (19). Consistent with this, we show that *Adipoq* expression is increased within the bone marrow of CKD mice. However, despite the decrease in iWAT and gWAT mass during CKD onset, it cannot be ruled out that WAT, due to increased production and secretion, is a contributary source to the increased circulating adiponectin in the CKD mice. This, however, is unlikely as in CR, adiponectin expression and secretion from WAT does not increase. Regardless of the source, adiponectin may, through autocrine mechanisms, promote preadipocyte differentiation, but whether such a ‘feed-forward’ mechanism contributes to BMAT accumulation in CKD is currently unclear (48).

Leptin is produced by WAT and the decrease in circulating levels in CKD mice are a likely consequence of reduced food intake and decreased body fat (49). As leptin-deficient *ob/ob* mice present with increased BMAT and decreased BMD, it is possible that low circulating leptin levels contribute to the BMAT accumulation and bone loss noted in the CKD mice (50). However, CR in female mice can increase BMAT without causing hypoleptinemia, whereas CR of male rabbits leads to decreased systemic leptin without BMAT expansion (19). Thus, it is possible that hypoleptinemia *per se* is neither necessary nor sufficient to promote BMAT expansion in CKD. In addition to adipokines, cytokines such as RANKL are also secreted from BMAds and we show that expression of *Tnfsf11* expression, but not *Tnfrsf11b*, is increased in CKD mice. Thus, BMAT-derived RANKL may also contribute to the bone loss noted in these mice (51).

The decrease in leptin levels is likely responsible for the increased circulating corticosterone in CKD mice (49, 52) and this observation is consistent with the phenotype of calorie restricted mice and Cushing’s disease, where glucocorticoid excess may contribute to BMAT expansion (19, 53). It remains unclear if increased corticosterone in CKD mice contributes to their BMAT accumulation and/or impaired bone health. Indeed, our pair-feeding studies suggest that CR *per se* is not responsible for BMAT accumulation in the CKD mice, suggesting that the mechanisms underlying BMAT expansion likely differ between CKD and CR.

In conclusion, we induced CKD-MBD in mice by feeding them an adenine-supplemented diet for up to 5-weeks. These mice lose both cortical and trabecular bone and accumulate adipocytes within the marrow cavity. BMAT accumulates prior to trabecular bone loss and is negatively correlated with trabecular bone volume fraction, suggesting that BMAT accumulation may contribute to bone loss in CKD. Although the numbers of adipocyte and osteoblast progenitor cells are unchanged during CKD onset, BMSCs from CKD mice have greater expression of adipogenic markers, suggesting enhanced adipogenic potential *in vivo* that may contribute to the increased BMAT in this disease.

These findings will inform future studies into the causes and consequences of BMAT accumulation in CKD, which may help to identify the cellular mechanisms involved and more specifically determine if the expansion of BMAds is at the expense of impaired osteoblast bone formation. Such studies are likely to uncover improved strategies for the clinical management of this condition.

## Data Availability

The original contributions presented in the study are included in the article/**Supplementary Files**, further inquiries can be directed to the corresponding author/s.
