# Supplementary material for: Bone marrow adipose tissue expansion and bone loss in experimental chronic kidney disease is independent of altered bone marrow stromal cell lineage determination
Source: Front Endocrinol (Lausanne). 2025 Sep 22;16:1666681. doi: 10.3389/fendo.2025.1666681 (PMC12497628; doi:10.3389/fendo.2025.1666681)

## Slide 1
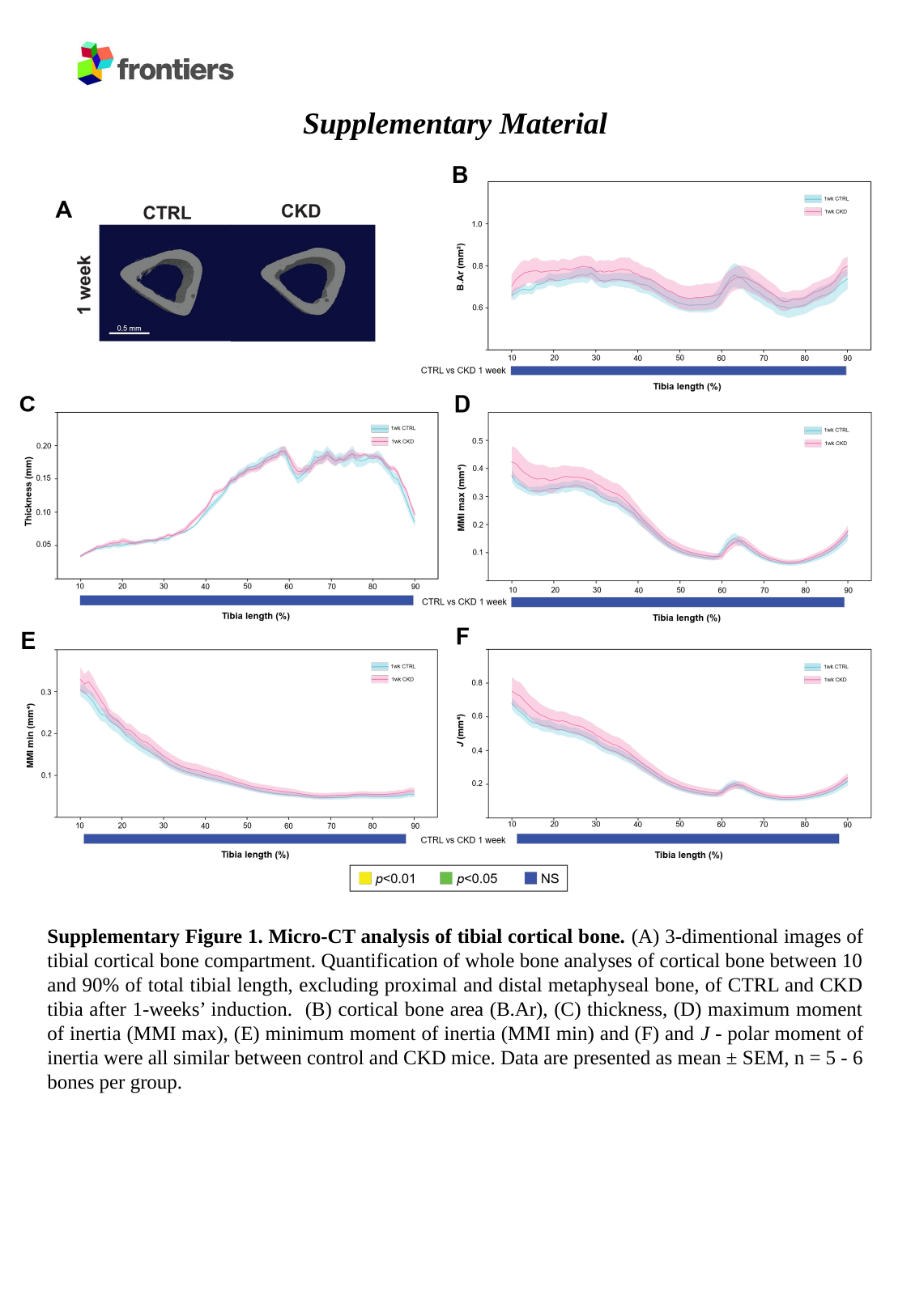

Supplementary Material
Supplementary Figure 1. Micro-CT analysis of tibial cortical bone. (A) 3-dimentional images of tibial cortical bone compartment. Quantification of whole bone analyses of cortical bone between 10 and 90% of total tibial length, excluding proximal and distal metaphyseal bone, of CTRL and CKD tibia after 1-weeks’ induction. (B) cortical bone area (B.Ar), (C) thickness, (D) maximum moment of inertia (MMI max), (E) minimum moment of inertia (MMI min) and (F) and J - polar moment of inertia were all similar between control and CKD mice. Data are presented as mean ± SEM, n = 5 - 6 bones per group.

## Slide 2
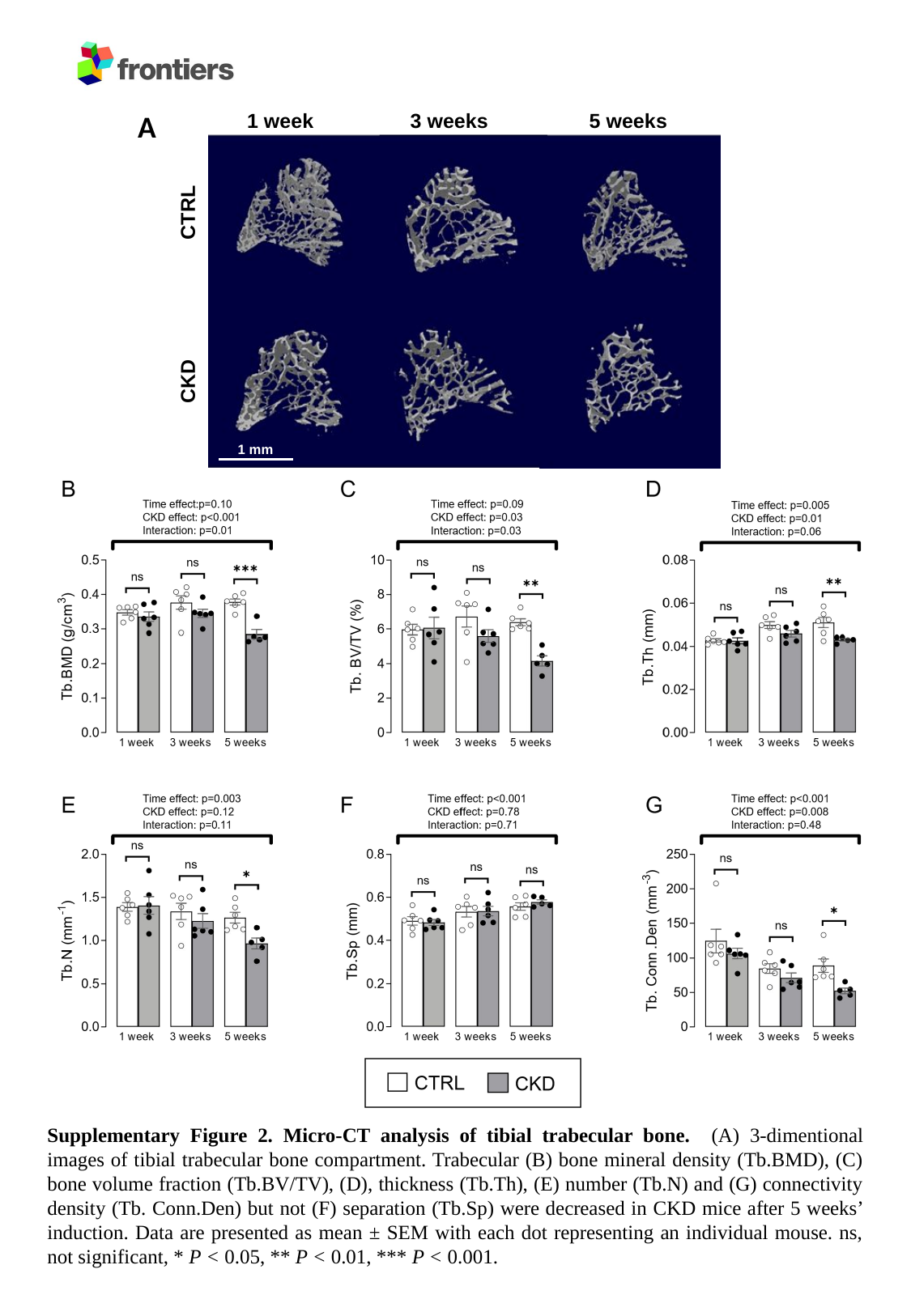

A
5 weeks
3 weeks
1 week
CTRL
CKD
1 mm
Supplementary Figure 2. Micro-CT analysis of tibial trabecular bone. (A) 3-dimentional images of tibial trabecular bone compartment. Trabecular (B) bone mineral density (Tb.BMD), (C) bone volume fraction (Tb.BV/TV), (D), thickness (Tb.Th), (E) number (Tb.N) and (G) connectivity density (Tb. Conn.Den) but not (F) separation (Tb.Sp) were decreased in CKD mice after 5 weeks’ induction. Data are presented as mean ± SEM with each dot representing an individual mouse. ns, not significant, * P < 0.05, ** P < 0.01, *** P < 0.001.

## Slide 3
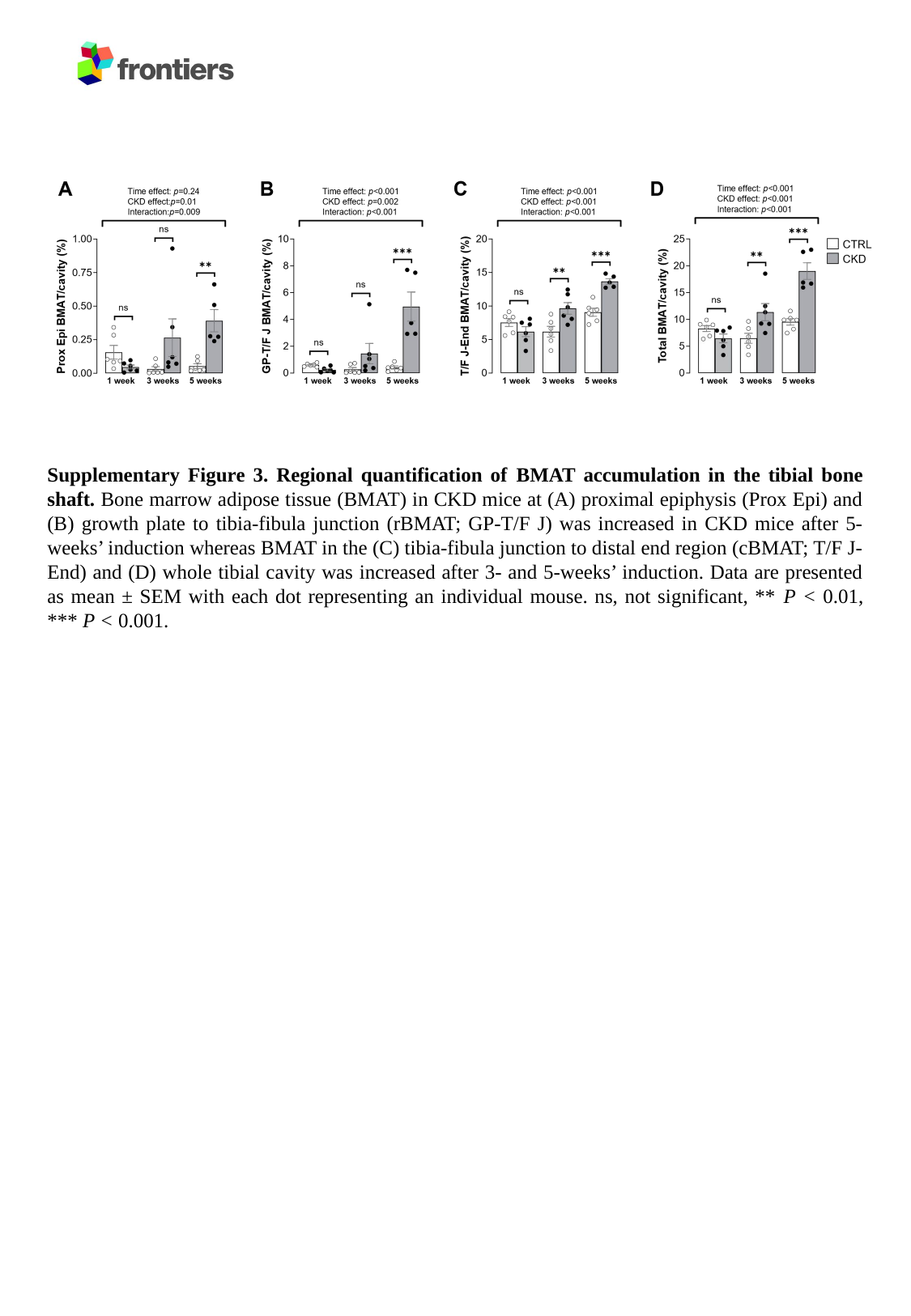

Supplementary Figure 3. Regional quantification of BMAT accumulation in the tibial bone shaft. Bone marrow adipose tissue (BMAT) in CKD mice at (A) proximal epiphysis (Prox Epi) and (B) growth plate to tibia-fibula junction (rBMAT; GP-T/F J) was increased in CKD mice after 5-weeks’ induction whereas BMAT in the (C) tibia-fibula junction to distal end region (cBMAT; T/F J-End) and (D) whole tibial cavity was increased after 3- and 5-weeks’ induction. Data are presented as mean ± SEM with each dot representing an individual mouse. ns, not significant, ** P < 0.01, *** P < 0.001.

## Slide 4
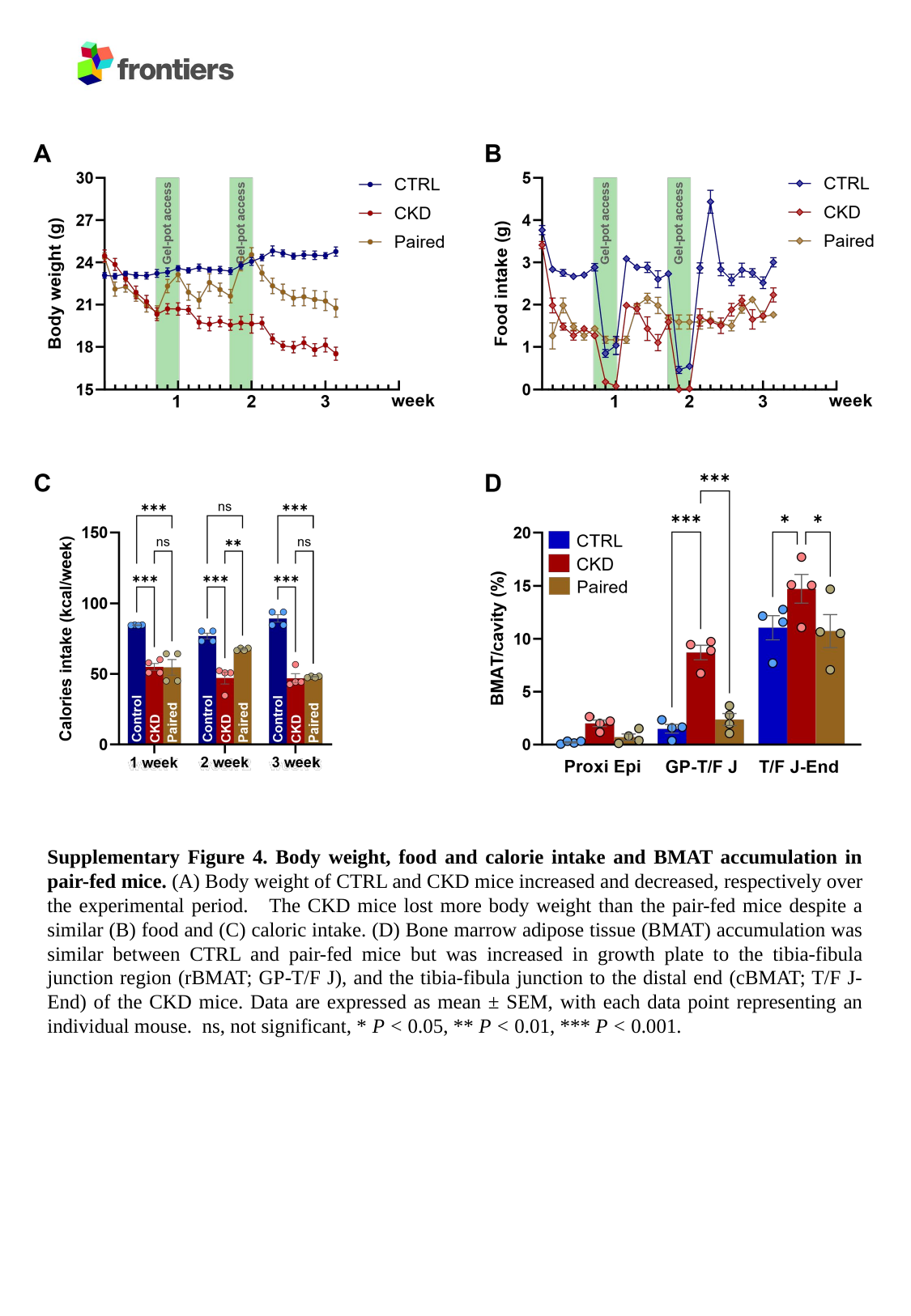

Supplementary Figure 4. Body weight, food and calorie intake and BMAT accumulation in pair-fed mice. (A) Body weight of CTRL and CKD mice increased and decreased, respectively over the experimental period. The CKD mice lost more body weight than the pair-fed mice despite a similar (B) food and (C) caloric intake. (D) Bone marrow adipose tissue (BMAT) accumulation was similar between CTRL and pair-fed mice but was increased in growth plate to the tibia-fibula junction region (rBMAT; GP-T/F J), and the tibia-fibula junction to the distal end (cBMAT; T/F J-End) of the CKD mice. Data are expressed as mean ± SEM, with each data point representing an individual mouse. ns, not significant, * P < 0.05, ** P < 0.01, *** P < 0.001.

## Slide 5
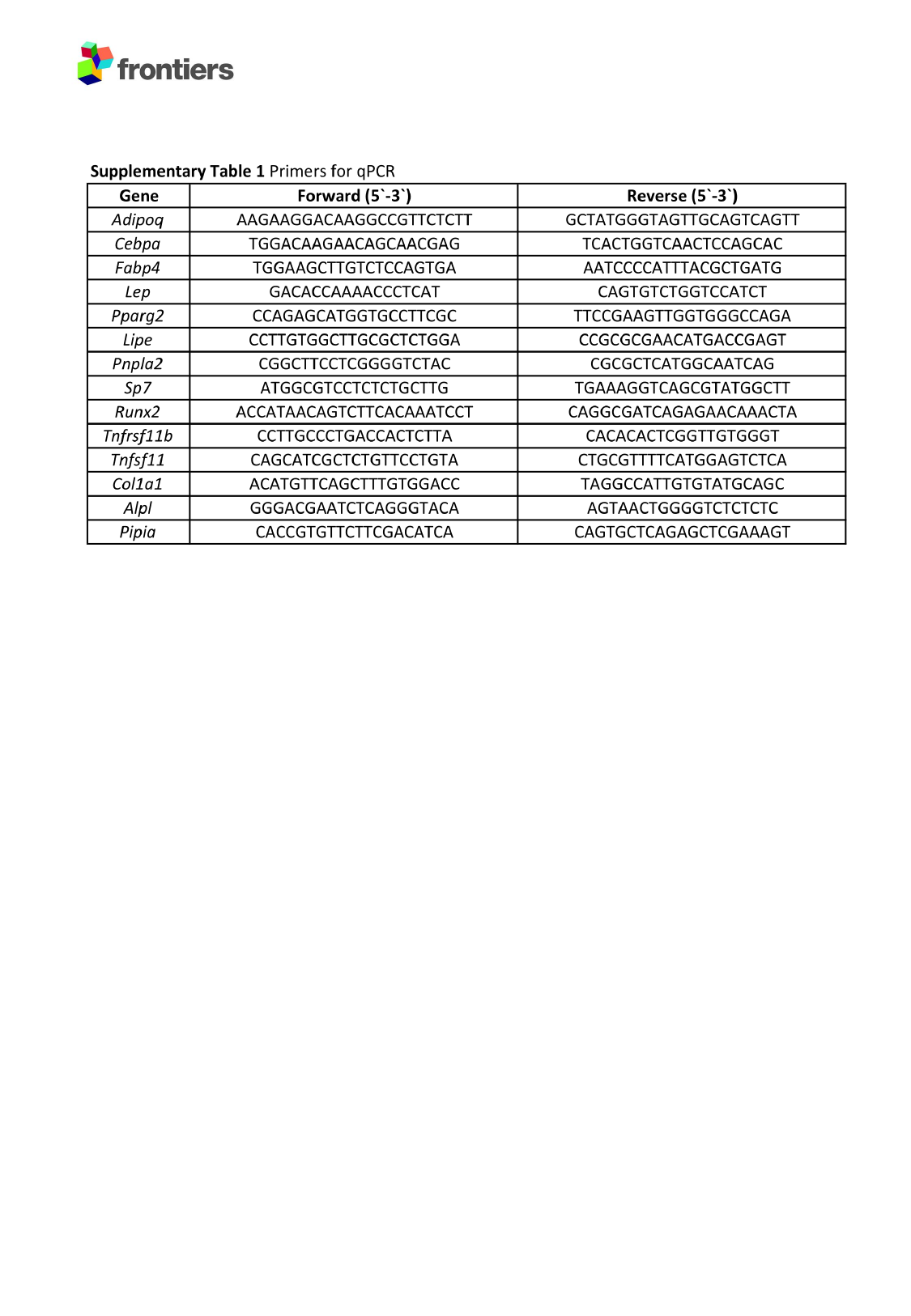

Supplement: Supplementary file 1 [file SupplementaryFile1.pptx]
